# Is Palpation Sufficient for Estimation of IOP Immediately Following Cataract Surgery?

**Published:** 2020-03-30

**Authors:** Andrew J Polk, Van Nguyen, John Jarstad

**Affiliations:** 1 University of Missouri School of Medicine, Department of Ophthalmology

**Keywords:** Palpation, Barraquer tonometer, Tono-Pen, Intraocular pressure, IOP; Accuracy, Micro-incision cataract surgery, Postoperative

## Abstract

The aim of this study was to evaluate the accuracy of standard palpation techniques and Barraquer tonometry relative to Tono-Pen for measurement of postoperative intraocular pressure (IOP) immediately following routine micro-incision cataract surgery (MICS). We conducted a prospective comparative analysis of postoperative IOP immediately after MICS in a single academic outpatient surgery center. A random block of 166 eyes that underwent MICS at our institution was selected for inclusion. Exclusion criteria consisted of any complications including posterior capsule rupture. IOP was measured immediately postoperatively, first with palpation or a Barraquer tonometer, then with a Tono-Pen handheld applanation tonometer. Measurements obtained by each method were compared. The mean difference between IOP measurements obtained by palpation and Tono-Pen was 10 mmHg, 95% confidence interval (CI; 8, 12); whereas the mean difference between IOP measurements obtained by Barraquer tonometer and Tono-Pen was 2 mmHg, 95% CI (1, 3). IOP measurements acquired via palpation differed from their corresponding Tono-Pen measurements by > 5 mmHg in 48.0% of cases compared to only 5.9% of measurements acquired using a Barraquer tonometer. Spearman correlation coefficient for measurements obtained by standard palpation and Tono-Pen was r = 0.397 (p < 0.01) compared to r = 0.774 (p < 0.01) for those obtained by Barraquer tonometer and Tono-Pen. In conclusion, palpation is not an accurate method for estimating IOP immediately after cataract surgery compared to Tono-Pen. Appropriate measurement and adjustment of IOP at the end of cataract surgery may decrease complications such as cystoid macular edema. In settings where a Tono-Pen is not readily available, Barraquer tonometry may serve as a reasonable and cost-effective alternative.

## INTRODUCTION

Cataract is the single most common cause of reversible blindness, responsible for 40% of blindness worldwide [1, 2]. In 2014, the World Health Organization estimated that there were 95 million people affected by cataracts worldwide [3]. It was estimated in 2000 that there were 20.5 million adults in the United States (US) over the age of 40 with cataracts (17.2% of this population) and it was predicted that this number would increase to 30.1 million by 2020 [4]. Cataract surgery is the most common and cost-effective procedure for the treatment of cataracts in many countries [5]. In the US, approximately 3 million cataracts are removed annually, making cataracts surgery one of the most commonly performed procedures [6]. It is well known that advanced age is a significant risk factor for cataract formation, [7] and with the increasing age of the United States population, it is likely that cataract surgery is going to become more prevalent in the foreseeable future.

In those patients undergoing surgery for cataracts, major complications are relatively rare, thanks in part to significant advances in surgical technique [8]. Even still, some of the most common complications of cataract surgery can result in significantly decreased vision, which can have considerable consequences with respect to patients’ quality of life. Some of the most common postoperative complications of cataract surgery include transiently elevated intraocular pressure (IOP), posterior capsule opacification, and cystoid macular edema (CME) [3, 9, 10]. In particular, CME is the number one cause of reduced vision and poor visual results following uncomplicated cataract surgery [2, 11, 12]. The prevalence of clinical CME following uncomplicated cataract surgery has been reported at 1.2-11%. When diagnosed using Optical Coherence Tomography, prevalence of CME following uncomplicated cataract surgery has been reported as high as 5-14% [3]

Given the increasing frequency at which cataract surgery is being performed, it is of great importance that surgeons minimize the risk of complications following this procedure. At the end of each cataract procedure, surgeons typically estimate IOP by palpating the cornea with a blunt surgical instrument to ensure that IOP is reasonably close to a physiologic value. This practice has gone unchallenged for years, with the understanding that “close” is probably good enough, but there is evidence to suggest that hypotony or ocular hypertension at the end of cataract surgery is associated with increased rates of postoperative complications like CME [9]. Therefore, it seems that surgeons should use more accurate methods to determine IOP in the operating theater. The goal of this study was to compare the relative accuracy of various methods for measuring IOP at the end of cataract surgery and to determine whether palpation alone is truly “good enough.”

## MATERIALS and METHODS

This was a prospective comparative analytic study performed at a single site from April 2016 to October 2016. Inclusion criteria consisted of any patient undergoing routine micro-incision cataract surgery (MICS). Exclusion criteria consisted of any intraoperative complications such as posterior capsule rupture. 

Following complete preoperative ocular examination, 160 eyes that underwent routine MICS entered the study. Of those eyes initially selected for inclusion, none met the exclusion criteria. All MICS procedures were performed by 2 experienced attending surgeons (each with 20,000+ cases) and 4 experienced resident surgeons in training at an outpatient surgery center at University of Missouri. At the end of each procedure, the viscoelastic material was removed, balanced salt solution (BSS) was used to reform the anterior chamber, and absence of leakage from the corneal incisions was verified. IOP was then measured by two separate techniques, first using either a Barraquer tonometer or estimation by palpation with a blunt hook manipulator and second using a Tono-Pen AVIA handheld applanation tonometer (AO Reichert, Depew, NY, USA). The Tono-Pen measurements were used as a standard control as it was felt to be the most objective and reliable of the three methods for determining IOP. If the initial IOP was excessively high or low based on clinical judgement of the surgeon, IOP was adjusted by addition or removal of BSS with a sterile irrigating cannula and IOP was reassessed. This was repeated until IOP was found to be within an acceptable range. Only the initial, unadjusted postoperative measurements obtained by each method were recorded and compared for concordance as part of this study. All patients received standard postoperative management and clinical follow-up including ocular examinations one-day, one-week, and one-month postoperatively. 

Institutional review board approval was obtained from the University of Missouri. An informed consent was obtained from each patient for the MICS procedure as well as postoperative measurement and adjustment of IOP in the operating theater.

Statistical analysis was performed by calculating the mean absolute difference between the two measurement techniques used to determine IOP for each group. Spearman’s correlation coefficients (r_s_) were also determined by plotting all IOP measurements acquired by palpation and Barraquer tonometer against their corresponding IOP acquired by Tono-Pen. All statistical analyses were conducted using IBM SPSS Statistics for Windows, version 24 (IBM Corp, Armonk, NY, USA).

## RESULTS

In total, 166 eyes were included in this study. A summary of patient demographics is presented in Table 1.

**Table 1:** Baseline Demographics of the Study Subjects

**Age (Y), mean (SD)**	67.8 ± 9.2
**Gender, % (n)**	
** Male**	42.2 (70)
** Female**	57.8 (96)
**Ethnicity, % (n)**	
** Caucasian**	77.7 (129)
** Black**	14.5 (24)
** Asian**	5.4 (9)
** Hispanic**	2.4 (4)

Abbreviations: SD: standard deviation; Y: years; n: number; %: percentage.

Postoperative IOP was estimated using standard palpation technique with a blunt hook manipulator in 98 cases (59%) and it was measured with a Barraquer tonometer for the remaining 68 cases (41%). These measurements were compared to postoperative IOP subsequently measured in all 166 cases using a Tono-Pen digital handheld tonometer.

For the palpation group, the mean estimated IOP was 22.7 mmHg (SD = 6.7) compared to 25.7 mmHg (SD = 15.7) measured by Tono-Pen. For the Barraquer group, the mean IOP was 18.9 mmHg (SD = 5.1) compared to 18.2 mmHg (SD = 4.7) measured by Tono-Pen.

**Figure 1:**
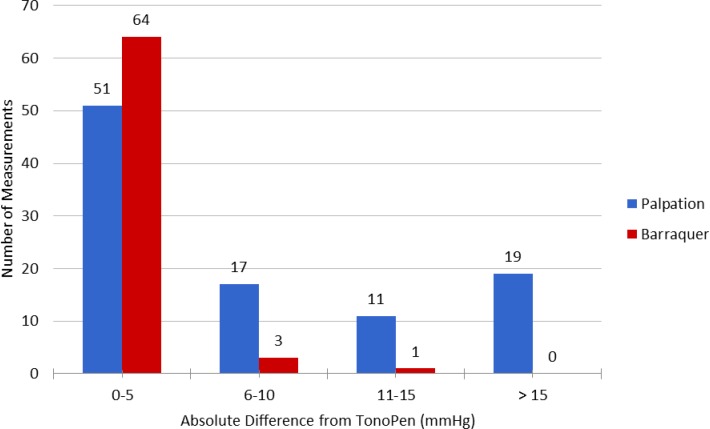
Histogram Demonstrating Distribution of the Absolute Difference Between IOP Measurements Obtained by Palpation and Barraquer Relative to Tono-Pen

**Figure 2:**
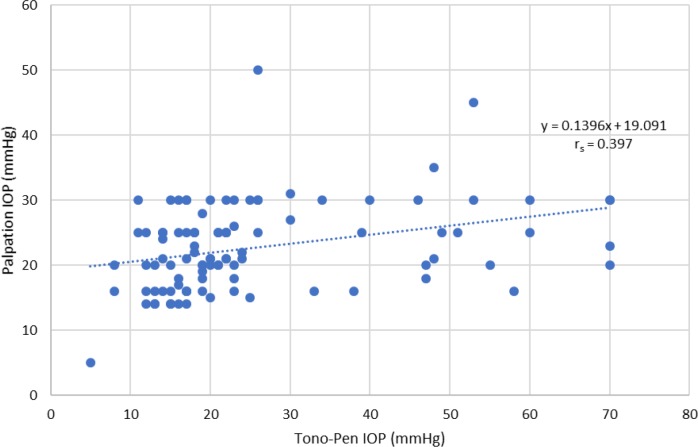
Low Concordance of IOP Measurements Obtained by Palpation and Tono-Pen Demonstrated by Trendline Slope and Spearman Correlation Coefficient Far From Equal to 1. In General, Surgeons Tend to Overestimate Low Extremes and Underestimate High Extremes of IOP When Relying on Palpation. mmHg: Millimetre of Mercury

**Figure 3:**
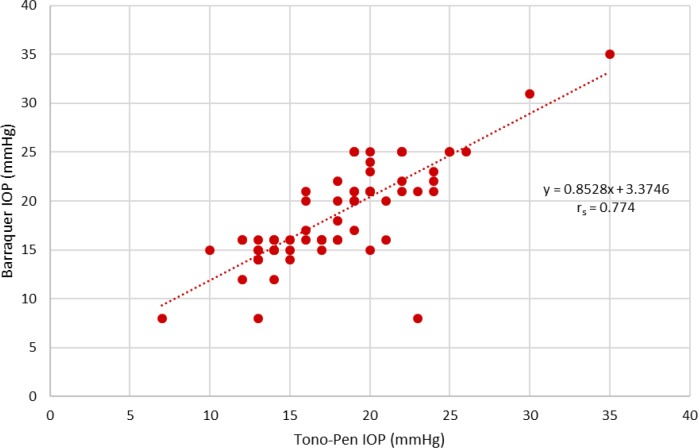
High Concordance of IOP Measurements Obtained by Barraquer and Tono-Pen Demonstrated by Trendline Slope and Spearman Correlation Coefficient Approximately Equal to 1. mmHg: Millimetre of Mercury

The mean absolute difference between IOP measurements obtained by palpation and Tono-Pen was 10 mmHg (95% CI [8,12], SD = 12); whereas, the mean absolute difference between IOP measurements obtained by Barraquer tonometer and Tono-Pen was 2 mmHg (95% CI [1, 3], SD = 2). IOP measurements acquired via palpation differed from their corresponding Tono-Pen measurements by > 10 mmHg in 30.6% (n = 30) of cases compared to only 1.5% (n = 1) of measurements acquired using a Barraquer tonometer (Figure 1). Spearman’s correlation coefficient for measurements obtained by palpation and Tono-Pen was r_s_ = 0.397 (p < 0.01) compared to r_s_ = 0.774 (p < 0.01) for measurements obtained by Barraquer tonometer and Tono-Pen (Figures 2 and 3). The Spearman’s correlation coefficient represents the strength of a monotonic relationship between sets of paired data points with the strongest relationships having a value of r_s_ = ±1. In general, an absolute value of r_s_ < 0.40 represents a “weak” relationship, whereas an absolute value of r_s_ ≥ 0.60 represents a “strong” relationship. Thus, these results suggest a higher rate of concordance between IOP measurements obtained by Barraquer tonometer and Tono-Pen than those obtained by palpation and Tono-Pen.

## DISCUSSION

There was a much higher rate of concordance between measurements obtained by Barraquer and Tono-Pen than those by palpation and Tono-Pen. These findings suggest that Barraquer tonometry is a significantly more accurate method of determining IOP than palpation. This conclusion is strongly supported by the results of a similar study by Anato and Kasaby, who found that digital assessment of IOP can be misleading [13].

However, these results are in contention with other studies declaring that digital assessment of IOP by palpation is relatively accurate, particularly when performed by experienced surgeons [14-16]. Clearly, the literature on this point is controversial. However, some of these studies found that accuracy of IOP estimates by palpation was influenced by several factors. One group observed significant variability in accuracy between surgeons, but also found that experience, lid edema, and tenderness of the globe influenced the accuracy of IOP estimates by palpation following penetrating keratoplasty [15]. It seems logical that accuracy of IOP estimates might vary from surgeon to surgeon, but it is hard to predict that factors like lid edema or globe tenderness would play a role. With this in mind, there could be other unpredictable factors influencing the reliability of IOP estimates by palpation. Without knowing these factors in advance, it may be difficult to predict the specific scenarios in which IOP estimates by palpation is appropriate.

The exact etiology of CME is not completely understood [12, 17, 18]. Some hypothesize that it may be related to postoperative inflammation. However, it is also thought that CME may be associated with mechanical forces [19]. It has been suggested that abnormal immediate postoperative intraocular pressure (IOP) may be a commonly overlooked risk factor for development of CME following routine cataract surgery procedures, particularly hypotony, but it may also occur with increased IOP [9]. In a previous study at our institution, it was found that patients with low IOP in the immediate postoperative period had significantly increased incidence of CME. Similarly, patients with elevated IOP had significantly increased incidence of macular thickening [9]. 

Given that our previous study demonstrated an increased incidence of CME with abnormal immediate postoperative IOP, we suggest that surgeons use a reliable tool such as a Tono-Pen or Barraquer tonometer to verify that IOP is within the physiologic range at the end of each cataract procedure. This allows necessary IOP adjustments to be made prior to leaving the operating theater and might decrease the risk of complications like pseudophakic CME. Other benefits of accurately verifying IOP in the operating theater include significant cost savings as fewer patients require IOP adjustments for unacceptable pressure spikes postoperatively [9]. These patients often require pressure-lowering medications, additional drops, tapping of the incision, and longer follow-up. All of these factors may incur significant expenses in terms of raw dollars, physician and staff time, and supplies [9] Furthermore, Barraquer tonometers are relatively inexpensive and can be reused for multiple cases ($380 for 15-21 mmHg Barraquer tonometer from Ocular Instruments vs. $3995 for Tono-Pen AVIA from Western-Ophthalmics or Lombart at the time of this article). This low cost would likely make Barraquer tonometers particularly useful for surgery in low-resource clinics where more expensive tools like a Tono-Pen cannot be easily obtained. We found both Barraquer tonometer and Tono-Pen to be convenient tools which did not require a great deal of time or effort to use in the operating theater.

Limitations of this study included a lack of data regarding postoperative complications since information pertaining to patient follow-up was not recorded. Such information would have no bearing on our final conclusions regarding relative accuracy of techniques for determining IOP in the immediate postoperative period. However, it would have been interesting to know whether adjustment of IOP at the end of these procedures resulted in a similar reduction in rates of CME as was demonstrated in our previous study. Another potential limitation of this study was the inherent inaccuracy of IOP measurements obtained by Tono-Pen. Although Goldmann applanation tonometry is generally considered the gold standard for measurement of IOP, the required equipment is too unwieldy for practical use in the operating theater and therefore could not be used as a control in this study [20]. The strength of this study was introduction of more accurate methods for determining IOP in the operating theater following MICS.

Future studies are recommended to clarify the relationship between IOP in the immediate postoperative period and the incidence of postoperative complications including CME. One study found that dedicated training with a cadaveric eye model could potentially improve an inexperienced surgeon’s ability to accurately estimate IOP by palpation [16]. It would be interesting for future studies to explore whether surgeons’ ability to accurately estimate IOP via palpation similarly improves over time with repeated use of the Tono-Pen as a training tool.

## CONCLUSION

Palpation may not be an accurate method for estimating IOP immediately after cataract surgery, a time when systematic measurement and adjustment of IOP may decrease complications such as CME. In settings where a Tono-Pen is not readily available, Barraquer tonometry may serve as a reasonable and cost-effective alternative. In addition to patient safety benefits, there may be significant economic advantages associated with accurate adjustment of IOP in the operating theater. The benefits of using a Tono-Pen or Barraquer tonometer to verify IOP at the end of cataract surgery strongly outweigh any inconvenience or expense that they may incur. Finally, we think this study makes a strong case that consistent use of these tools should be a standard practice in the operating theater.

## DISCLOSURE

Ethical issues have been completely observed by the authors. All named authors met the International Committee of Medical Journal Editors (ICMJE) criteria for authorship of this manuscript, took responsibility for the integrity of the work as a whole, and gave final approval for the version to be published. No conflict of interest was presented. Funding/Support: None. The datasets analyzed during this study are available from the corresponding author on reasonable request.
